# Resilience, plasticity and robustness in gene expression during aging in the brain of outbred deer mice

**DOI:** 10.1186/s12864-021-07613-2

**Published:** 2021-04-21

**Authors:** E Soltanmohammadi, Y Zhang, I Chatzistamou, H. Kiaris

**Affiliations:** 1 Department of Drug Discovery and Biomedical Sciences, College of Pharmacy, University of South Carolina, SC Columbia, USA; 2 Department of Pathology, Microbiology and Immunology, School of Medicine, University of South Carolina, SC Columbia, USA; 3 Peromyscus Genetic Stock Center, University of South Carolina, SC Columbia, USA

**Keywords:** Deer mice, Outbred, Transcriptome, Expression correlation, P. leucopus, P. maniculatus, Aging

## Abstract

**Background:**

Genes that belong to the same network are frequently co-expressed, but collectively, how the coordination of the whole transcriptome is perturbed during aging remains unclear. To explore this, we calculated the correlation of each gene in the transcriptome with every other, in the brain of young and older outbred deer mice (P. leucopus and P. maniculatus).

**Results:**

In about 25 % of the genes, coordination was inversed during aging. Gene Ontology analysis in both species, for the genes that exhibited inverse transcriptomic coordination during aging pointed to alterations in the perception of smell, a known impairment occurring during aging.

In P. leucopus, alterations in genes related to cholesterol metabolism were also identified. Among the genes that exhibited the most pronounced inversion in their coordination profiles during aging was THBS4, that encodes for thrombospondin-4, a protein that was recently identified as rejuvenation factor in mice. Relatively to its breadth, abolishment of coordination was more prominent in the long-living P. leucopus than in P. maniculatus but in the latter, the intensity of de-coordination was higher.

**Conclusions:**

There sults suggest that aging is associated with more stringent retention of expression profiles for some genes and more abrupt changes in others, while more subtle but widespread changes in gene expression appear protective. Our findings shed light in the mode of the transcriptional changes occurring in the brain during aging and suggest that strategies aiming to broader but more modest changes in gene expression may be preferrable to correct aging-associated deregulation in gene expression.

## Introduction

Genes that belong to the same transcriptional networks have expression profiles that are correlated [1, 2]. Stress-inducing stimuli inflict changes in gene expression in a manner that is highly coordinated. During stress as well as in pathology, novel transcriptional networks emerge while others cease to exist, a process that collectively implies transcriptional reprogramming and aims to induce adaptive changes to attain homeostasis [3–7]. Several strategies have been developed to evaluate gene co-expression, especially during disease development [8]. These approaches primarily focus on the identification of specific gene modules that exhibit differential co-expression patterns at the states under comparison, without directly conveying information about the extent of (de-) coordination at the whole transcriptome level.

The robustness by which transcriptional networks adapt and operate under adverse conditions is thought to reflect the ability of the corresponding tissues to cope with stress [9–11]. To that end, the magnitude of the changes in expression of specific transcripts is considered indicative for the alterations characterizing pathology, with the overall and collective breadth of the alterations receiving minimal attention [12–14]. For example, how widespread such changes in gene expression are during aging, whether they are more intense in pathology and at which extent they disrupt the operation of established transcriptional networks remain unclear [15–18].

It is plausible that by comparing the correlation of each gene in the transcriptome with every other gene, in two experimental conditions such as aging groups, may provide evidence regarding the magnitude, the mode and the intensity of the transcriptomic changes that occur. Indeed, by focusing on the unfolded protein response we have shown before, that during aging as well as in metabolic pathology, despite the minimal changes in expression of individual genes, changes in the coordination profile of the UPR are more tightly linked to pathology [19–22]. Beyond the UPR, we have also demonstrated that the coordination profile of highly expressed genes is also linked to frailty syndrome in people [23].

Such analyses can be especially pertinent in studying tissues from genetically diverse experimental animals. In outbred species, variation in expression profiles among individuals may restrict the power of the differential expression analyses, however it empowers investigations focusing on gene expression correlations, since the latter rely on the diversity of the experimental measurements. In the present study we evaluated the mode of coordination in the brain transcriptome of outbred rodents (Peromyscus), that reportedly differ in their lifespan and exhibit differential propensity for neurodegenerative changes during aging [24–26]. Specifically, we assessed the correlation in the expression in each gene in the transcriptome with every other gene, and then we tested how this correlation changes during aging. Gene Ontology (GO) studies were also performed to unveil biological processes related to the genes that exhibited inversion of transcriptome coordination in the aging group.

Focal for this analysis is the assessment of the composite correlation index (Pearson’s composite, Pc) that is defined for each transcript and reflects the correlation of each of the individual correlation coefficients for a given gene with the whole transcriptome, between two experimental conditions [23]. To that end, minimal changes in correlation coefficients and retention of the mode of coordination in younger and older animals will result in highly positive Pc values, which in turn are indicative of higher robustness in the profile of gene expression. Conversely, changes in gene expression between the experimental groups, will lead to reduction of the Pc values and will be indicative of more extensive transcriptional reprogramming and plasticity in gene expression. By applying this strategy in genetically diverse individuals from two outbred Peromyscus species we were able to record how extensively their brain transcriptome is rearranged during aging. Furthermore, by comparing the changes recorded in a species that is more prone to neurodegeneration versus another that appears more resistant, we were able to appreciate the relative role of plasticity and robustness in the transcriptomic profiles during aging and which mode is linked more, to the emergence of pathology. Finally, we were able to unveil biological processes that are impacted by such emerging coordination patterns and to identify specific genes, that despite the fact that their overall expression is not highly altered during aging, their co-expression profiles with the whole transcriptome is highly perturbed.

## Results

### Brain degenerative changes of deer mice during aging

**Fig. 1:**
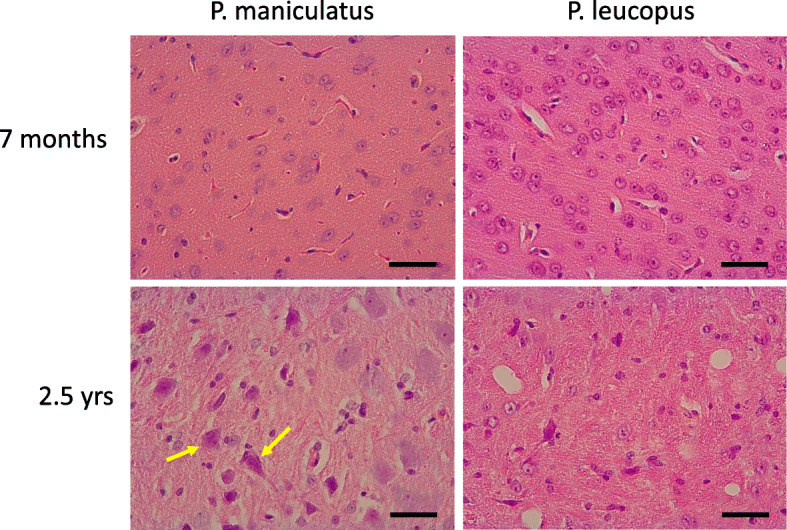
Brain histology in young and older P. maniculatus and P. leucopus. H&E stained sections are shown. Arrows indicate neurons exhibiting evidence of neurodegeneration. Scale barσ: 40 µΜ

Recent studies indicated that in P. maniculatus, lesions consistent with degenerative changes were detectable in the brain of older animals [19]. These lesions, that were also shown to exhibit positivity in Congo Red for amyloid deposits, were practically absent from P. leucopus individuals of similar age [19]. By comparing the histology of brain tissues from *P. maniculatus* and *P. leucopus* we were able to identify several neurons with dense eosinophilic cytoplasm and dark nuclei in the former, in individuals sacrificed at about 25–29 months. These lesions that were present in all animals tested are consistent with degenerative changes (Fig. 1).

### P. maniculatus and P. leucopus exhibit distinct expression coordination profiles during aging

**Fig. 2:**
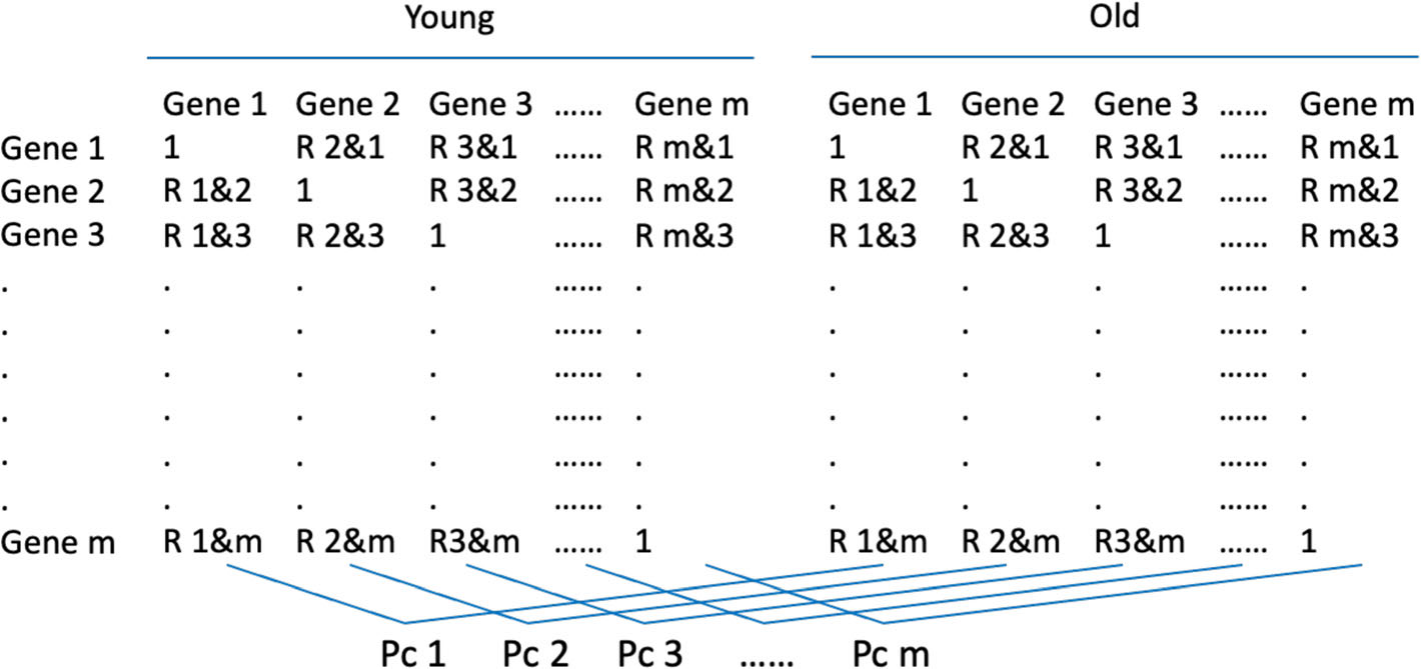
Diagrammatic depiction of the steps followed for the calculation of the composite correlation index (Pc). Person’s correlation R values of each gene with all other genes were calculated in samples of young and old animals respectively. Then the Pc value was calculated as the Person’s R of all R values of each gene between young and old animal samples

RNAseq was performed in the brain of *P. leucopus* and *P. maniculatus* from younger (about 7 months old) and older (about 25–29 months old) animals and revealed 19,718 and 15,262 unique transcripts (data deposited as GSE166394). To test how the transcriptome in each species is coordinated during aging we calculated the Pc index as follows (Fig. 2): Initially we calculated the correlation coefficient (R, Pearson’s) for each transcript with every other transcript in the transcriptome, for each age group. This analysis produced 19,718 and 15,262 unique R values, for *P. leucopus* and *P. maniculatus* respectively, for each age group. The Pc for each transcript reflected its composite correlation coefficient of all correlation coefficients calculated before, for the given transcript, between the two age groups. Therefore, high Pc values indicate that the mode of coordination between young and aged specimens is retained for the given gene, while lower Pc values indicate abolishment of coordination. Conversely, negative Pc values suggest that the profile of coordination is inversed during aging.

**Fig. 3:**
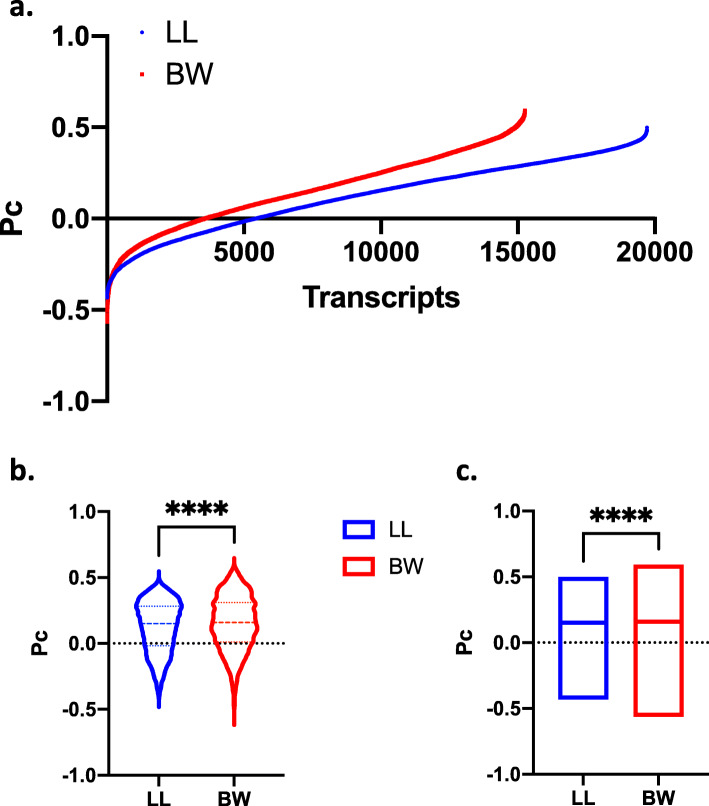
Pc calculation for the transcriptome of P. leucopus (LL) and P. maniculatus (BW). Scatter plots of Pc versus transcripts are shown in (**a**), box and violin plots depicting Pc distribution are shown in (**b**) and bar plots showing the median values are shown in (**c).** In all graphs the actual number of transcripts surveyed in each transcriptome are shown. ****, *P* < 0.00001

**Fig. 4:**
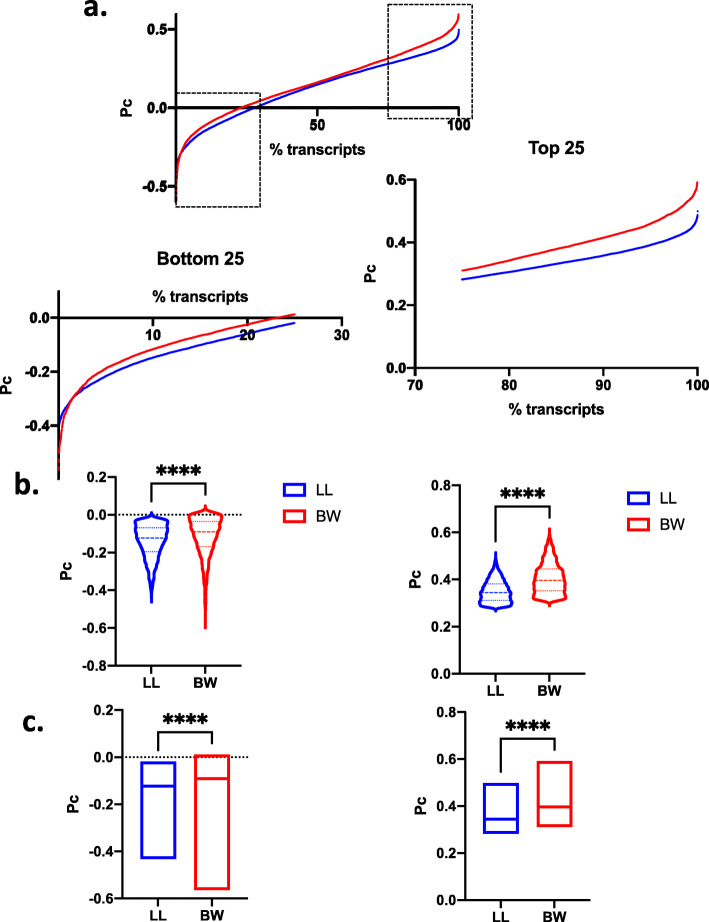
Pc calculation for the transcriptome of P. leucopus (LL) and P. maniculatus (BW). Values were normalized versus the total number of transcripts surveyed. Scatter plots of Pc versus transcripts are shown in (**a**). In dashed brackets the top and bottom 25th percentile are shown and magnified in the plots shown below. Box and violin plots are shown in (**b**) and bar plots showing the median values are shown in (**c)** for the bottom (left) and top (right) 25th percentile respectively. ****, *P* < 0.00001

As shown in Fig. 3, in both species the majority of the transcripts exhibited positive Pc values, suggesting that the mode of coordination was retained between the young and older animals, for most of the genes. *P. maniculatus* (BW stock) consistently exhibited higher Pc values than *P. leucopus* (LL stock) [average Pc(BW) = 0.15 vs. Pc(LL) = 0.12, *P* < 0.0001] suggesting that the correlation in gene expression, at the whole transcriptome level, was retained more prominently in the former species, as compared to the latter. Thus, *P. leucopus*, were subjected to more extensive reprogramming than *P. maniculatus*, as indicated by the fact that more transcripts exhibited lower degree of correlation in expression with the whole transcriptome, between the younger and the older animals. These differences persisted and remained statistically significant (*P* < 0.00001) even when transcript numbers were normalized for their difference in the overall number of transcripts surveyed (Fig. 4). Following such normalization, 23 and 27 % of the transcripts for *P. maniculatus* and *P. leucopus* respectively exhibited negative Pc values indicating inversion of the coordination profile during aging.

Yet, despite the fact that fewer transcripts were collectively analyzed for P. maniculatus as compared to P. leucopus, the range of Pc values unveiled was wider in the former species, both towards the negative and the positive ends of the distribution (Fig. 4). This suggests that in P. maniculatus a higher number of transcripts, stringently retains its correlation profile (highly positive Pc values, Fig. 4c) during aging or is subjected to more drastic inverse changes (highly negative Pc values, Fig. 4b). Conversely, the transcriptome of P. leucopus during aging is subjected to more widespread changes (lower Pc on average, Fig. 4a) but the corresponding values were tighter (more restricted range towards both positive and negative ends, Fig. 4b).

### Genes exhibiting higher inversion in coordination during aging

Gene ontology (GO) analysis for the top 500 genes with the highest negative Pc value in both species, pointed to changes in the perception of smell during aging (Table 1). For P. leucopus, changes influencing alcohol and lipid metabolism were also predicted (Table 1).

**Table 1:** Gene Ontology analyses the genes exhibiting the lowest Pc values during aging in both species

GO biological process complete	Fold Enrichment	Raw P-value (Fisher)	FDR correction
**P. leucopus**			
secondary alcohol biosynthetic process (GO:1,902,653)	20.75	2.92E-09	2.30E-05
cholesterol biosynthetic process (GO:0006695)	20.75	2.92E-09	1.53E-05
sterol biosynthetic process (GO:0016126)	19.42	6.56E-10	1.03E-05
alcohol biosynthetic process (GO:0046165)	8.29	8.58E-07	1.93E-03
steroid biosynthetic process (GO:0006694)	8.28	2.44E-07	7.69E-04
secondary alcohol metabolic process (GO:1,902,652)	7.74	4.09E-08	1.61E-04
cholesterol metabolic process (GO:0008203)	7.31	7.65E-07	2.01E-03
sterol metabolic process (GO:0016125)	6.76	1.56E-06	3.06E-03
organic hydroxy compound biosynthetic process (GO:1,901,617)	5.1	1.91E-05	2.73E-02
alcohol metabolic process (GO:0006066)	4.01	9.72E-06	1.53E-02
sensory perception of smell (GO:0007608)	0.07	6.18E-06	1.08E-02
**P. maniculatus**			
sensory perception of smell (GO:0007608)	0.12	1.04E-07	1.64E-03

**Fig. 5:**
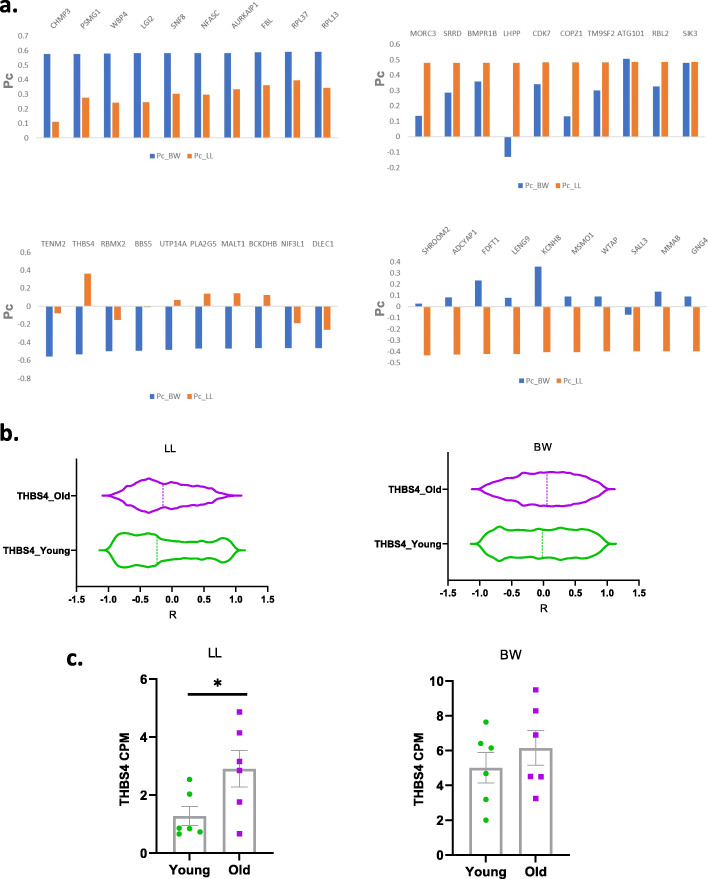
Genes exhibiting retention or inversion of coordination profiles during aging in P. leucopus (LL) and P. maniculatus (BW). The top 10 genes with highest Pc index in P. maniculatus are shown in (**a**) upper left and in LL shown in (**a**) upper right. The top 10 genes with lowest Pc index in BW are shown in (**a**) lower left and in LL shown in (**a**) lower right. Violin plots depicting the Pearson’s R value distribution of THBS4 associated transcriptome in young and older LL and BW are shown in (**b**). The relative expression of THBS4 in young and older LL and BW are shown in (**c**). *, *P* < 0.05

The genes that exhibited the highest Pc values, either positive or negative for each species, are shown in Fig. 5a (upper panel). In general, highly positive Pc values exhibited the same trend in both species. The only exception was LHPP that while it remained highly positive in P. leucopus (Pc = 0.48), in P. maniculatus it reverted to moderately negative (Pc=-0.13). LHPP encodes for a histidine phosphatase that has been shown to function as a tumor suppressor [27].

As opposed to the top positively coordinated transcripts that showed similar mode of correlation in both species, the top negatively coordinated transcripts exhibited more drastic changes in one species than in the other, at which Pc remained positive (Fig. 5a, lower panel). More pronounced was the behavior of THBS4 (thrombospondin-4) that in P. maniculatus had a Pc=-0.53 while in P. leucopus Pc = 0.36. This suggests that while in P. leucopus the coordination profile of THSB4 with the whole transcriptome persisted during aging, in P. maniculatus it reverts to negative, implying that transcripts that in young animals had positive correlation display negative correlation in the aged specimens (Fig. 5b). Vice versa, the transcripts that had negative correlation with THSB4 in young P. maniculatus had acquired positive correlation in the older animals (Fig. 5b). The relative expression of THBS4 cannot account for this difference since in older P. leucopus its levels increased almost twofold, while in P. maniculatus its levels remained similar in younger and older animals (Fig. 5c).

## Discussion

Conventionally, the interrogation of the transcriptome for the detection of aging-associated alterations is performed by differential expression analyses that aim to identify specific, deregulated transcripts and point to strategies for therapeutic intervention. Nevertheless, beyond the relative levels of expression, how the whole transcriptome is coordinated may also be of special value in explaining age-related changes. To that end, it is plausible that the expression of specific transcripts may be retained but their relative abundance as compared to other transcripts may change, causing cumulatively drastic changes in expression profiles and ultimately affecting functionality. Targeted strategies focusing in either the UPR, or others, exploring only the highly expressed genes in the transcriptome, supported these notions suggesting that beyond levels of expression, coordination of transcripts may be of special value in various conditions and pathologies [19, 20, 22].

In order to test these hypotheses at the level of the whole transcriptome in an unbiased manner, we evaluated how the coordination profile of the whole brain transcriptome changes during aging. Thus, we calculated for the correlation coefficient of each individual gene expressed, and every other gene in the transcriptome. Subsequently, a composite Pearson’s correlation coefficient (Pc) was calculated comprising of the correlation of these correlation coefficients, for each and every gene in the transcriptome, in young versus older animals. We applied this analysis to two highly related deer mouse species, *P. leucopus* (LL stcock) and *P. maniculatus* (BW stock). P. leucopus has a reported lifespan of about 8 years while P. maniculatus lives about 4 years. The analysis involved young animals, about 7 months old and older animals, about 25–29 months old. Between the two, neurodegenerative changes were detected primarily in the older P. maniculatus. Furthermore, distinct profiles of DNA methylation have been reported in aging in these two species that while for P. leucopus, differentially methylated genes indicated changes in angiogenesis, in P. maniculatus they were more consistent with the regulation of gamma delta T cells, a process that is associated with neurodegeneration [28, 29].

Earlier studies involving multiple tissues from inbred C57BL/6 mice indicated that during aging a decline in gene expression correlation occurs in a modular as opposed to a uniform manner, with NF-kB target genes being most prominently influenced [30]. According to this analysis a reduction, by 26 %, was found in network edges of the older mice, that reflect the correlation in expression between two genes. This is comparable to the results of our present analysis that showed that 23 and 27 % of the transcripts for *P. maniculatus* and *P. leucopus* respectively had negative Pc values, which in turn points to the inversion of the coordination profile during aging. Furthermore, the two deer mouse species evaluated here displayed distinct profiles of coordination in their brain transcriptome during aging: In *P. leucopus*, Pc was significantly lower than in *P. maniculatus*, implying more extensive reorganization of the transcriptome during aging. As regards though to the magnitude of the changes recorded, reflected to the lower and higher ends of the Pc values’ distribution, they appeared higher in *P. maniculatus*. Thus, in this species, the changes although affected a smaller fraction of the transcriptome, they were more abrupt (producing higher Pc values, being either negative or positive).

Whether these changes are related to the fact that *P. leucopus* reportedly exhibits prolonged lifespan as compared to *P. maniculatus* and the fact that the latter exhibits more pronounced evidence of neurodegenerative changes in the older animals, cannot be formally supported. Furthermore, in the absence of detailed evaluation of the corresponding lesions by using specific markers these histological alterations may reflect deterioration of the brain tissue that is irrelevant to the typical neurodegeneration as described in people. Nevertheless, it leads to the intriguing hypothesis that reduced robustness and increased plasticity of the transcriptome is linked to resilience to aging. Consistently with this notion, pharmacological interventions aspiring to alleviate aging, it is probably preferrable to seek for strategies aiming to cause wider yet more subtle changes in expression profiles, as opposed to those that may constitute “correction” strategies that target intensively specific networks.

Of note is that in both deer mouse species, the genes that underwent the most potent inversion of coordination profiles predicted changes in the perception of smell which validates the present approach since loss of smell represents a known impairment that accompanies aging [31, 32]. Furthermore, it underscores the impact of neural dysfunction in the loss of smell at aging, beyond the changes occurring in the olfactory epithelium over time. In P. leucopus, processes associated with lipid metabolism were suggested that may reflect adaptive responses due to the aging-associated loss of lipid content in the brain [33].

Among the transcripts that exhibited most pronounced changes in their coordination profile, THBS4 is of interest since in *P. leucopus* it retains a highly positive correlation in young and old animals (Pc = 0.36), but in *P. maniculatus* it reverted to highly negative (Pc=-0.53). This gene encodes for thrombospondin-4 that has been linked to neurodegeneration before and was identified recently as a rejuvenation factor in mice [34, 35].

A limitation of the present analysis is that whole brain tissue sections were used. Thus, it remains formally possible that different brain regions with distinct expression profiles may exhibit different coordination patterns which in turn, may interfere with the present analysis. Nevertheless, even if expression levels change across different anatomical locations, coordination patterns of gene networks are likely retained. An additional limitation is related to the number of specimens used per group (*n* = 6), therefore, the results should be interpreted with caution.

Collectively the present study shows that in a long living deer mouse species, the transcriptome is subjected to broader yet more subtle changes, as compared to a species that develops some neurodegeneration and reportedly has a more restricted lifespan and at which transcriptomic changes are less broad but more intense. Thus, strategies aiming to extensive but less severe changes in the transcriptome may be preferrable over those that incur more restricted changes, of increased however intensity. Whether these findings are relevant to other species including humans and characterize other pathologies as well, remains to be established.

## Materials and methods

### Animals

 We obtained animals from the Peromyscus Genetic Stock Center (PGSC), University of South Carolina (USC), Columbia, SC (RRID:SCR_002769). North American deer mice, P. maniculatus bairdii (BW stock) is a closed colony bred in captivity since 1948 and descends from 40 ancestors wild-caught near Ann Arbor, Michigan. White footed mice (P. leucopus, LL stock) is a colony derived from 38 wild ancestors captured between 1982 and 1985 near Linville. The animals were divided into two groups of six animals each, of young (7 months old for both species) and old (25–29 months old for P. maniculatus and 28 months old for P. leucopus) animals in each species at the time we sacrificed them. All experiments were approved by the Institutional Animal Care and Use Committee (IACUC) and the Department of Health and Human Services, Office of Laboratory Animal Welfare, University of South Carolina (Approval No. 2349-101211-041917).

### Histology

Brain tissue was harvested from the animals at the indicated ages, fixed in 4 % neutral buffered formalin and subsequently embedded in paraffin. Tissues sections were stained with Hematoxylin and Eosin (H&E) by using standard protocols and histology analysis was performed.

### RNA isolation and analysis

RNA was extracted from brain tissue by using the RNeasy Mini Kit (Qiagen) according to the manufacturer’s instructions. RNA yield was evaluated by spectrophotometric absorbance (NanoDrop™ 2000/2000c Spectrophotometers Thermo Scientific™) at 260 nm (A260) and the purity was evaluated at 260/280 nm (A260/280). RNA was eluted into 250ng/µl of nuclease-free water, tested for RNA integrity assay and subjected to RNA sequencing. RNA integrity was assessed using the Agilent Bioanalyzer and samples had a quality score ≥ 9.6. RNA libraries were prepared using established protocol with NEBNext Ultra II Directional RNA Library Prep Kit for Illumina (NEB). RNA sequencing was performed as described [23, 36]. One sample was analyzed from each of the 6 animal per group (24 samples in total). Sequences were aligned to the P. maniculatus and P. leucopus reference genome using STAR v2.7.2b [37] with default parameters. Reads were counted using the featureCounts function of the Subreads package [38] in R using Gencode M6 GTF and summarized at exon, transcript, or gene level. The counts were normalized with counts per million (CPM) method and used for correlation analysis.

### Coordination analysis

The procedure of the correlation analysis is shown in Fig. 2. The Person’s correlation R values of each gene with all other genes in the whole transcriptome were calculated in samples of young and old *P. maniculatus* and *P. leucopus*, respectively. The composite correlation (Pc) index was calculated as the Person’s R of all R values of each gene between young and old animals. This transformation assigned a unique Pc value to each of these genes which reflects the degree by which coordination with the whole transcriptome changes between young and old animals for the corresponding genes of interest. All calculations were conducted with R 3.6.3. Gene Ontology analysis was applied to the top 500 genes exhibiting the lowest Pc values, using Mus annotation [39, 40].

## Data Availability

RNA seq data have been submitted to NCBI (GSE166394). Peromyscus animals are available from the Peromyscus Genetic Stock Center.
